# Supplementary material for: Isolation and Characterization of the First Temperate Virus Infecting Psychrobacillus from Marine Sediments
Source: Viruses. 2022 Jan 8;14(1):108. doi: 10.3390/v14010108 (PMC8779076; doi:10.3390/v14010108)
Supplement: Supplementary file 1 [file viruses-14-00108-s001.zip › Supplementary materials.pdf]

Supplementary materials

Table S1. Primers used in this study.

Name	Sequence
16S-27 F	5'-AGAGTTTGATCCTGGCTCAG-3'
16S-1492 R	5'-GGTTACCTTGTTACGACTT-3'
M13-F	5'-TGTAAAACGACGGCCAGT-3'
M13-R	5'-CAGGAAACAGCTATGACC-3'
PV1-attL F	5'-AGGGTCTTCTAACGCATCAATCA-3'
PV1-attL R	5'-TTAGGAGAACCAATAGCGGTGTA-3'
PV1-attR F	5'-TGCAGGAATAAGATTCGAGTGC-3'
PV1-attR R	5'-AACGAGGTTCGTAATATGTTTGG-3'
PVJ1-ORF75 F	5'-CAACCCATCTAACCAAATTCCATA-3'
PVJ1-ORF75 R	5'-TGACAGTCGTAATTGCCACCC-3'
PVJ1-39 F	5'-AATATTGTATCCATCACTACCACCACAG-3'
PVJ1-13562 R	5'-GCATAACGGCAAGAAAATCAAATTCGAAT-3'
PVJ1-13292 F	5'-ATCGATGGATTTCGTCTTTGTGATATTT-3'
PVJ1-26177 R	5'-CTATTAGAGAAATTGGAGAAGCGGTGG-3'
PVJ1-25817 F	5'-TGGATGATTAGGTATTTCAAACAATCGTGT-3'
PVJ1-37067 R	5'-AAAACAGGCAAGCGTGAAGTTTTTATTG-3'
PVJ1-36731 F	5'-TCAATTAAACTCATCAGATCACCTTCACT-3'
PVJ1-49091 R	5'-CGATACGTGAAAATCATCCATGGTACAC-3'
PVJ1-48703 F	5'-TCGATTGCCATTGCTCTAAACTTCTCTAG-3'
PVJ1-778 R	5'-AAAAAGCAGGTTACGAATATCCCAGTATC-3'
PVJ1-qPCR F	5'-GATTTGAGGTAAGTCGGCTAAGAG-3'
PVJ1-qPCR R	5'-TAGGAGAAGAAGGGTACTTGTGGA-3'

Table S2. Predicted ORFs of PVJ1.

ORF no. (orientation)	Position	Predicted function	BLASTP hit(s) (accession no.; E value)	Length (No. of residues)	Predicted TMH
1(-)	1234-1401	hypothetical protein	hypothetical protein JCM9152_2062 [*Bacillus hemicellulosilyticus* JCM 9152] (GAE30 648.1;2e-08)	55	0
2(-)	1658-2770	hypothetical protein	hypothetical protein [*Bacillus* sp. 522_BSPC] (WP_048716486.1;2e-140)	370	0
3(-)	2826-3215	hypothetical protein	hypothetical protein [*Psychrobacillus lasiicapitis*] (WP_142537303.1;3e-80)	129	0
4(-)	3241-3411	hypothetical protein	hypothetical protein HMPREF1210_02984 [*Paenisporosarcina* sp. HGH0030] (EPD50136.1;1e-12)	56	2
5(-)	3451-3942	Single-stranded DNA-binding protein A	single-stranded DNA-binding protein [*Psychrobacillus lasiicapitis*] (WP_142538361.1; 4e-101)	163	0
6(-)	3939-4154	hypothetical protein	hypothetical protein [*Psychrobacillus* sp. *Z8*] (WP_142640942.1;2e-36)	71	0
7(-)	4201-4395	hypothetical protein	no significant hit	64	0
8(-)	4459-5769	DNA cytosine methyltransferase	DNA cytosine methyltransferase [*Planomicrobium* sp. MB-3u-38] (WP_101802793.1;0)	436	0
9(-)	5786-5932	hypothetical protein	no significant hit	48	0
10(-)	5929-6273	hypothetical protein	hypothetical protein [*Bacillus mycoides*] (WP_063226329.1;3e-42)	114	0

11(-)	6263-6478	hypothetical protein	hypothetical protein CHI07_17015 [*Paenibacillus* sp. 7884-2] (PAE27897.1;2e-29)	71	0
12(-)	6515-7027	Holliday junction resolvase RecU	Holliday junction resolvase RecU [*Psychrobacillus* sp. FJAT-21963] (WP_056828373.1;3e-108)	170	0
13(-)	7024-7209	hypothetical protein	hypothetical protein C7437_1011045 [*Psychrobacillus insolitus*] (PZX07923.1;1e-25)	61	0
14(-)	7196-7363	hypothetical protein	hypothetical protein [*Psychrobacillus* sp. FJAT-21963] (WP_056828380.1;2e-13)	55	0
15(-)	7367-7984	dUTPase	dUTPase [*Psychrobacillus lasiicapitis*] (WP_142538354.1;5e-143)	205	0
16(-)	7981-8844	putative RNA polymerase sigma 70 factor	hypothetical protein [*Psychrobacillus lasiicapitis*] (WP_142538353.1;2e-133)	287	0
17(-)	8846-9109	hypothetical protein	hypothetical protein [*Psychrobacillus* sp. FJAT-21963] (WP_056828384.1;9e-36)	87	0
18(-)	9106-9282	hypothetical protein	hypothetical protein [*Lysinibacillus mangiferihumi*] (WP_107896777.1;3e-07)	58	0
19(-)	9348-9515	hypothetical protein	hypothetical protein HMPREF1210_01171 [*Paenisporosarcina* sp. HGH0030] (EPD52791.1;0.002)	55	0
20(-)	9512-9652	hypothetical protein	pilus assembly protein PilO [*Bacillus* sp. OxB-1] (WP_144399717.1;4e-07)	46	0

21(-)	9645-9827	hypothetical protein	hypothetical protein C7437_1011051 [*Psychrobacillus insolitus*] (PZX07929.1;2e-19)	60	0
22(-)	9843-10673	replication initiator protein DnaA	ATP-binding protein [*Psychrobacillus insolitus*] (WP_111438536.1;2e-151)	276	0
23(-)	10612-11406	replication protein	replication protein [*Staphylococcus capitis*] (WP_096389332.1;2e-94)	264	0
24(-)	11422-11625	hypothetical protein	hypothetical protein [*Planomicrobium* sp. MB-3u-38] (WP_101801962.1;4e-09)	67	0
25(-)	11636-12337	Putative metallo-hydrolase YycJ	MBL fold metallo-hydrolase [*Psychrobacillus lasiicapitis*] (WP_142538349.1;1e-161)	233	0
26(-)	12337-13233	recombinase RecT	recombinase RecT [*Psychrobacillus lasiicapitis*] (WP_142538348.1;0)	298	0
27(-)	13233-13511	hypothetical protein	hypothetical protein [*Oceanobacillus profundus*] (WP_118888883.1;2e-27)	92	0
28(-)	13511-13756	hypothetical protein	hypothetical protein [*Psychrobacillus lasiicapitis*] (WP_142538347.1;5e-33)	81	0
29(-)	13758-15752	AAA family ATPase,putative nucleoside triphosphate hydrolase	AAA family ATPase [*Psychrobacillus* sp. FJAT-21963] (WP_056828403.1;0)	664	0
30(-)	15845-16054	hypothetical protein	hypothetical protein [*Psychrobacillus lasiicapitis*] (WP_142538345.1;3e-32)	69	0
31(-)	16057-16785	putative anti repressor	hypothetical protein [*Psychrobacillus* sp. FJAT-21963] (WP_056828410.1;8e-133)	242	0
32(-)	17137-17328	hypothetical protein	hypothetical protein [*Lysinibacillus sphaericus*] (WP_069512420.1;2e-23)	63	0

33(+)	17367-17555	XRE family transcriptional regulator	XRE family transcriptional regulator [*Bacillus megaterium*] (AUO14742.1;7e-15)	62	0
34(+)	17744-18100	XRE family transcriptional regulator	transcriptional regulator with XRE-family HTH domain [*Psychrobacillus insolitus*] (PZX07946.1;9e-57)	118	0
35(-)	18631-19803	site-specific integrase	site-specific integrase [*Lysinibacillus sphaericus*] (WP_069512418.1;0)	390	0
36(-)	20308-20829	hypothetical protein	hypothetical protein [*Lysinibacillus contaminans*] (WP_053583196.1;1e-36)	173	0
37(-)	20880-21101	hypothetical protein	hypothetical protein [*Lysinibacillus* sp. SYSU K30002] (WP_126657363.1;2e-12)	73	0
38(-)	21173-21376	hypothetical protein	no significant hit	67	0
39(-)	21411-21635	hypothetical protein	hypothetical protein [*Lysinibacillus* sp. SYSU K30002] (WP_126657362.1;2e-09)	74	0
40(-)	21632-22492	hypothetical protein	no significant hit	286	0
41(+)	22687-23181	hypothetical protein	hypothetical protein C7437_1011005 [*Psychrobacillus insolitus*] (PZX07883.1;7e-65)	164	0
42(+)	23186-23392	hypothetical protein	no significant hit	68	1
43(+)	23495-24208	stress protein	stress protein [*Kurthia zopfii*] (WP_109349344.1;1e-52)	237	1
44(-)	24229-24429	hypothetical protein	hypothetical protein [*Psychrobacillus* sp. OK032] (WP_093273641.1;1e-24)	66	0

45(-)	24446-24775	YolD-like family protein	YolD-like family protein [*Psychrobacillus* sp. FJAT-21963] (WP_056828286.1;8e-40)	109	0
46(-)	24788-26047	DNA polymerase V	UV damage repair protein UvrX [*Psychrobacillus lasiicapitis*] (WP_142538397.1;0)	419	0
47(-)	26044-26247	Transcription regulator LexA	winged helix-turn-helix transcriptional regulator [*Psychrobacillus lasiicapitis*] (WP_142538396.1;9e-27)	67	0
48(-)	26247-26468	putative transcriptional regulator	transcriptional regulator [*Psychrobacillus* sp. OK032] (WP_093271147.1; 9e-29)	73	0
49(+)	26594-27355	putative DNA ligase C1	hypothetical protein [*Psychrobacillus lasiicapitis*] (WP_142538395.1;4e-169)	253	0
50(-)	27397-28161	Lysozyme, N-acetylmuramoyl-L-alanine amidase	N-acetylmuramoyl-L-alanine amidase [*Psychrobacillus lasiicapitis*] (WP_142538394.1;6e-143)	254	0
51(-)	28155-28439	holin	phage holin [*Bacillus sporothermodurans*] (WP_066234733.1;5e-36)	94	2
52(-)	28456-28896	hypothetical protein	hypothetical protein [*Rummeliibacillus stabekisii*] (WP_066785529.1;1e-48)	146	1
53(-)	28957-29175	hypothetical protein	hypothetical protein [*Psychrobacillus lasiicapitis*] (WP_142539850.1;3e-39)	72	0
54(-)	29405-29629	hypothetical protein	hypothetical protein [*Bacillus jeotgali*] (WP_079504712.1;2e-04)	74	0
55(-)	29725-30150	hypothetical protein	hypothetical protein [*Bacillus* sp. FJAT-22090] (WP_144535422.1;1e-66)	141	1
56(-)	30356-31414	acyltransferase	acyltransferase [*Bacillus drentensis*] (WP_082797500.1;5e-64)	352	10

57(-)	31509-33509	polygalacturonase	hypothetical protein [*Psychrobacillus* sp. OK032] (WP_093266492.1;4e-22)	666	0
58(-)	33499-34524	putative portal protein	DUF2313 domain-containing protein [*Psychrobacillus* sp. OK032] (WP_093266494.1;6e-82)	341	0
59(-)	34539-34925	hypothetical protein	hypothetical protein [*Psychrobacillus* sp. OK032] (WP_093266496.1;1e-68)	128	0
60(-)	34941-35990	baseplate J	baseplate J/gp47 family protein [*Geobacillus stearothermophilus*] (WP_033016789.1;2e-106)	349	0
61(-)	35987-36394	hypothetical protein	DUF2634 domain-containing protein [*Sporosarcina* sp. ANT_H38] (WP_149582044.1;8e-63)	135	0
62(-)	36394-36804	hypothetical protein	DUF2577 domain-containing protein [*Paenisporosarcina* sp. HGH0030] (WP_081637782.1;3e-56)	136	0
63(-)	36744-37703	hypothetical protein	hypothetical protein [*Psychrobacillus* sp. FJAT-21963] (WP_056828316.1;0)	319	0
64(-)	37714-38400	LysM peptidoglycan-binding domain-containing protein	LysM peptidoglycan-binding domain-containing protein [*Psychrobacillus* sp. FJAT-21963] (WP_056828318.1;1e-122)	228	0
65(-)	38400-40673	tape measure protein	tape measure protein [*Psychrobacillus* sp. FJAT-21963] (WP_056828321.1;0)	757	5
66(-)	40936-41334	phage portal protein	phage portal protein [*Psychrobacillus* sp. FJAT-21963] (WP_056828323.1;4e-65)	132	0

67(-)	41352-41816	core tail protein	phage tail protein [*Psychrobacillus* sp. FJAT-21963] (WP_056828324.1;2e-91)	154	0
68(-)	41831-43150	sheath tail protein	phage tail protein [*Bacillus oleronius*] (WP_078109550.1;0)	439	0
69(-)	43150-43356	hypothetical protein	hypothetical protein [*Psychrobacillus* sp. FJAT-21963] (WP_056828328.1;7e-14)	68	0
70(-)	43540-43968	hypothetical protein	hypothetical protein [*Psychrobacillus* sp. FJAT-21963] (WP_056828329.1;3e-67)	142	0
71(-)	43952-44347	hypothetical protein	HK97 gp10 family phage protein [*Psychrobacillus* sp. FJAT-21963] (WP_056828331.1;2e-70)	131	0
72(-)	44347-44685	hypothetical protein	hypothetical protein [*Ammoniphilus oxalaticus*] (WP_120190023.1;2e-52)	112	0
73(-)	44705-45064	hypothetical protein	hypothetical protein [*Sporosarcina* sp. ANT_H38] (WP_149582056.1;1e-47)	119	0
74(-)	45057-45224	hypothetical protein	no significant hit	55	0
75(-)	45266-46309	major capsid protein	phage capsid protein [*Psychrobacillus* sp. FJAT-21963] (WP_056828339.1;0)	347	0
76(-)	46326-46685	head decoration protein	hypothetical protein [*Psychrobacillus* sp. FJAT-21963] (WP_056828341.1;3e-68)	119	0
77(-)	46700-47305	minor structural protein	chemotaxis protein [*Psychrobacillus* sp. FJAT-21963] (WP_056828343.1;4e-96)	201	0

78(-)	47432-47629	hypothetical protein	no significant hit	65	0
79(-)	47696-47911	putative baseplate-J protein	baseplate J/gp47 family protein [*Clostridium* sp. ASF356] (WP_004033628.1;2e-05)	71	0
80(-)	47943-48959	SPP1 family phage head morphogenesis protein	hypothetical protein [*Bacillus azotoformans*] (WP_003330933.1;9e-152)	338	0
81(-)	48952-50391	phage portal protein	phage portal protein [*Psychrobacillus* sp. FJAT-21963] (WP_056828351.1;0)	479	0
82(-)	50401-51723	terminase large subunit	PBSX family phage terminase large subunit [*Bacillus oleronius*] (WP_078109561.1;0)	440	0
83(-)	51701-52585	terminase small subunit	terminase small subunit [*Bacillus praedii*] (WP_131238737.1;6e-146)	294	0
84(-)	52709-53140	hypothetical protein	hypothetical protein [*Bacillus thuringiensis*] (WP_098830210.1;3e-46)	143	4

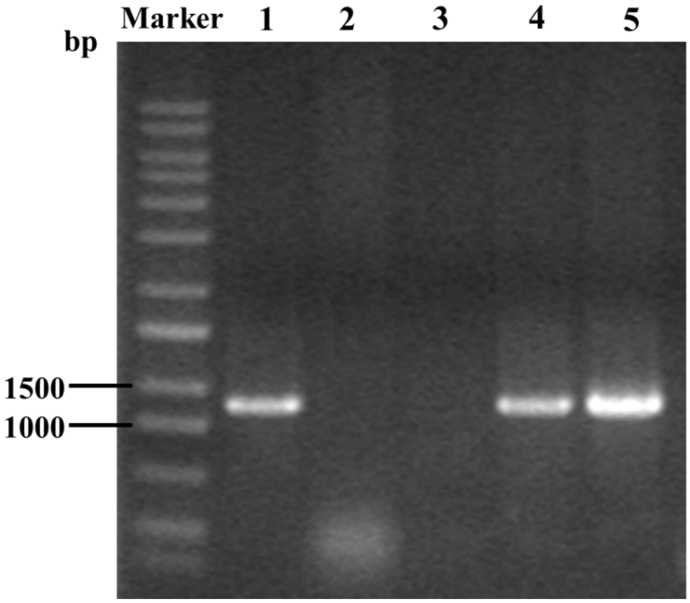

Figure S1. Detection of the PVJ1 genome sequence in the original hydrothermal sediment sample. Metagenomic DNA was prepared from sediment sample GC2, and used as the template for PCR reactions with the primer pair PVJ1-ORF75 F/R. The reaction products were analyzed by agarose gel electrophoresis. Lane 1, metagenomic DNA of GC2; lane 2, metagenomic DNA of SC; lane 3, deionized water; lane 4, genomic DNA of *Psychrobacillus* sp. GC2J1; lane 5, genomic DNA of PVJ1.

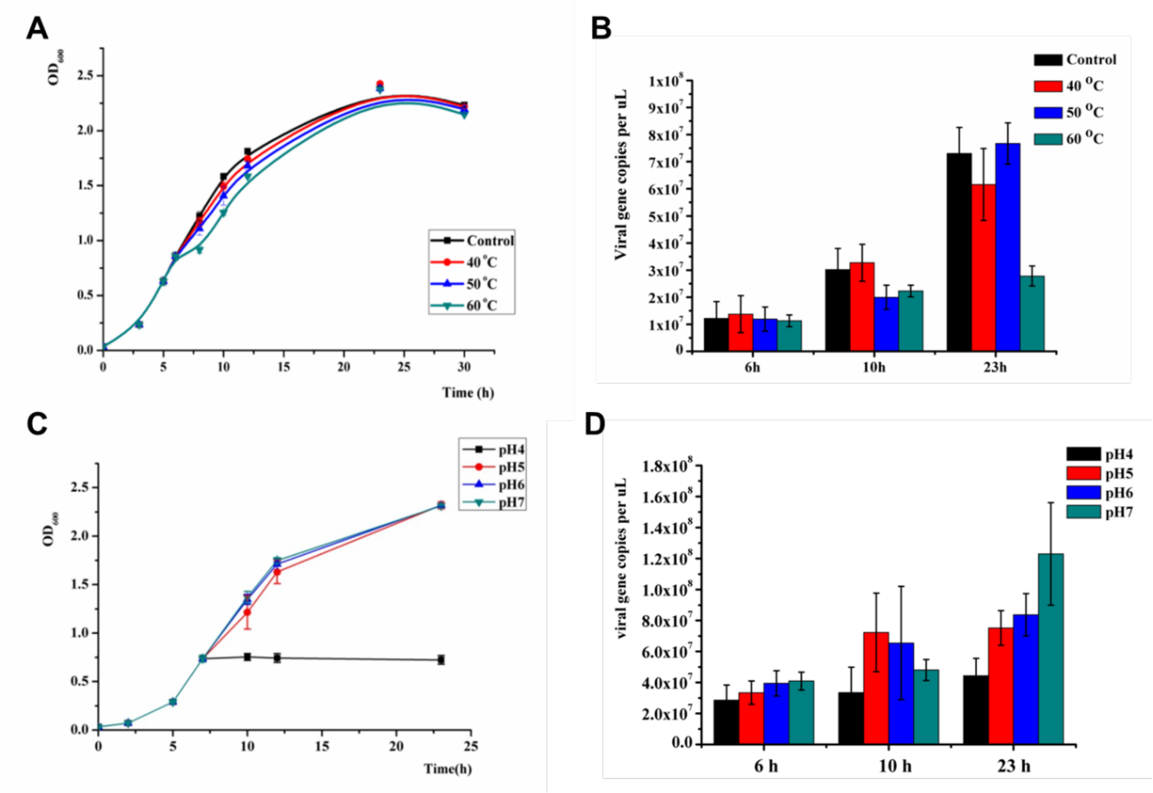

Figure S2. Effects of temperature and pH on the growth of *Psychrobacillus* sp. GC2J1 and the titer of PVJ1. GC2J1 was grown to an OD_{600} of ~0.8 at 25ºC and pH7. (A and B) The culture was heat-shocked for 10 min at indicated temperatures and returned to incubation at 25ºC. (C and D) The pH of the culture was adjusted to indicated values, and the incubation was continued. The growth of *Psychrobacillus* sp. GC2J1 (A and C) and the PVJ1 titer (B and D) were determined.

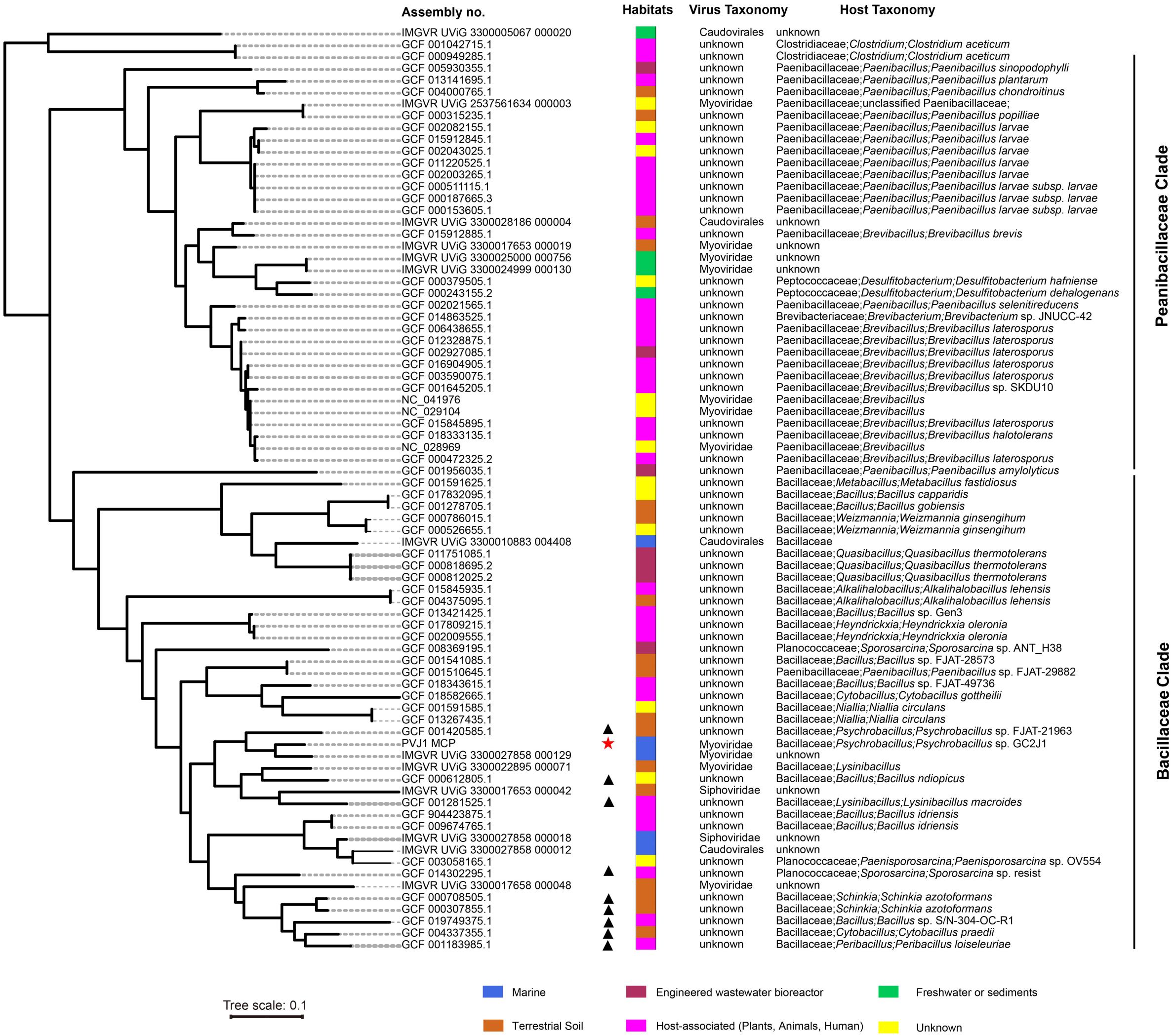

Figure S3. Maximum likelihood (ML) phylogenetic tree of the homologues of PVJ1 MCP. The tree was constructed based on the amino acid sequences of phage MCPs sharing ≧70% identity with PVJ1 MCP. The MCP of PVJ1 is marked with a red star. Phage genomes selected for synteny analysis (Figure 3) are shown by triangles. Habitats from which phage sequences were obtained are indicated. More information on the sequences is in Table S3.
